# The ASTUTE Health study protocol: Deliberative stakeholder engagements to inform implementation approaches to healthcare disinvestment

**DOI:** 10.1186/1748-5908-7-101

**Published:** 2012-10-22

**Authors:** Amber M Watt, Janet E Hiller, Annette J Braunack-Mayer, John R Moss, Heather Buchan, Janet Wale, Dagmara E Riitano, Katherine Hodgetts, Jackie M Street, Adam G Elshaug

**Affiliations:** 1 School of Population Health, The University of Adelaide, North Terrace, Adelaide, SA, Australia; 2 Faculty of Health Sciences, Australian Catholic University, 115 Victoria Parade, Fitzroy, VIC, Australia; 3 Australian Commission on Safety and Quality in Health Care, 1 Oxford Street, Darlinghurst, NSW, Australia; 4 Department of Health Care Policy, Harvard Medical School, 180 Longwood Ave, Boston, MA, USA; 5 The Commonwealth Fund, One East 75th Street, New York, NY, USA

**Keywords:** Public participation, User involvement, Disinvestment, Policy

## Abstract

**Background:**

Governments and other payers are yet to determine optimal processes by which to review the safety, effectiveness, and cost-effectiveness of technologies and procedures that are in active use within health systems, and rescind funding (partially or fully) from those that display poor profiles against these parameters. To further progress a disinvestment agenda, a model is required to support payers in implementing disinvestment in a transparent manner that may withstand challenge from vested interests and concerned citizens. Combining approaches from health technology assessment and deliberative democratic theory, this project seeks to determine if and how wide stakeholder engagement can contribute to improved decision-making processes, wherein the views of both vested and non-vested stakeholders are seen to contribute to informing policy implementation within a disinvestment context.

**Methods/design:**

Systematic reviews pertaining to illustrative case studies were developed and formed the evidence base for discussion. Review findings were presented at a series of deliberative, evidence-informed stakeholder engagements, including partisan (clinicians and consumers) and non-partisan (representative community members) stakeholders. Participants were actively facilitated towards identifying shared and dissenting perspectives regarding public funding policy for each of the case studies and developing their own funding models in response to the evidence presented. Policy advisors will subsequently be invited to evaluate disinvestment options based on the scientific and colloquial evidence presented to them, and to explore the value of this information to their decision-making processes with reference to disinvestment.

**Discussion:**

Analysis of the varied outputs of the deliberative engagements will contribute to the methodological development around how to best integrate scientific and colloquial evidence for consideration by policy advisors. It may contribute to the legitimization of broad and transparent stakeholder engagement in this context. It is anticipated that decision making will benefit from the knowledge delivered through informed deliberation with engaged stakeholders, and this will be explored through interviews with key decision makers.

## Background

### Disinvestment as a policy approach to healthcare safety and quality

As health system managers increasingly seek ways in which to improve quality of care and constrain resource use, the concept of health technology reassessment with potential for disinvestment has gained currency. Having existed for some time in the realms of business and manufacturing, the consideration of disinvestment practices within healthcare is relatively new, with antecedents in evidence-based medicine (EBM) and health technology assessment (HTA). Disinvestment seeks to improve health outcomes by evaluating existing health services, identifying those that do not provide safe, effective, or cost-effective care, and redirecting funding away from these services and towards those with superior safety, effectiveness, and cost-effectiveness profiles through a variety of policy approaches [1,2]. It does not by definition need to be a dichotomous choice to fund or not to fund; disinvestment can occur by degrees, whereby subsidies may be restricted to subgroups of patients for whom there is evidence of potential for benefit based on specific clinical characteristics (referred to as ‘refining the indications’ for service provision).

While there has been international interest in disinvestment, it remains an essentially theoretical construction that has only been operationalized in a limited manner in few jurisdictions [3]. A number of challenges are faced in developing and implementing the concepts of disinvestment into pragmatic policy approaches. While methods for the assessment of new and emerging technologies and services have become relatively well-established in the processes of HTA, governments and other payers are still to determine the optimal processes by which to review existing technologies and procedures. Often these pre-date the EBM and HTA era and thus have escaped rigorous evaluation of safety, effectiveness, and cost-effectiveness, yet are entrenched as offerings within healthcare.

In considering processes for refining funding for established technologies and services, contested elements may include: what is counted as ‘evidence’ and by whom; the ‘burden’ of evidence required; and the consultative components that may be necessary to support disinvestment decisions. A best-practice model to support payers in pursuing explicit efforts towards disinvestment in a manner that may withstand challenge from vested interests and interested citizens is yet to emerge [1].

With a view to bridging this divide between policy and practice, a competitively funded, Australian-based research project was established in 2009. The purpose of the ASTUTE Health study (*A*ssessing *S*ervice and *T*echnology *U*se *T*o *E*nhance Health) is to develop, trial, and evaluate a transparent process by which to refine the indications for services, to ensure investment in procedures with favourable safety, effectiveness, and cost-effectiveness profiles. Independent of government, this multidisciplinary project seeks to incorporate methods from HTA and deliberative democratic theory and, as such, represents a novel approach to the development of evidence-based health policy wherein the views of both vested and non-vested stakeholders are seen to contribute valuably and uniquely to informing policy decisions within a disinvestment context.

### Deliberative democracy: Involving the community

Deliberative democratic methods have gained prominence in recent years, driven in part by the rise of evidence-informed policy-making and the potential for these methods to assist in involving the broader community in democratic governance that extends beyond voting [4,5]. Current methods of policy development and decision making are considered to contain democratic deficits, created by the opaque, but often dominant roles of strong special interest groups/experts, an inability to represent heterogeneous public interests, and a lack of processes by which to encourage input from adequately informed members of the general public [6,7]. Additionally, there remains a need for ‘health services to be accountable to users as taxpayers, voters, and consumers’ [8].

Debate around ‘the fair and reasonable allocation of resources has become a prominent one in modern day discourses about healthcare’ [9]. Health funding decisions, particularly those which relate to some level of reimbursement retraction (more effective redistribution notwithstanding) may carry significant moral implications, be highly contested (by a range of stakeholders) and historically have not been made in an explicit and transparent manner. The complexity and contestability inherent in disinvestment decisions position such health policies as key candidates for the integration of deliberative methods in their construction [4].

Within this study, members of the community will be involved in making explicit the barriers and facilitators of disinvestment (with reference to both the specific case studies and the broader disinvestment context), and to determine whether stakeholder engagement could contribute to creating improved and transparent decision-making processes in this policy domain. Additionally, it is anticipated that the colloquial evidence collected from community members will enhance the scientific evidence base derived from the systematic reviews, and balance the perspectives of other more actively vested stakeholders and special interest groups.

### Experiential knowledge: involving vested stakeholders

In order to align this research project with the realities of policy making, to which a wide variety of stakeholders contribute, the perspectives, knowledge, and interests of vested stakeholders (clinical and consumer groups) will also be included in addition to the views of non-partisan citizens. This broad, evidence-informed engagement seeks to mimic and enhance the diverse range of inputs with which health policy decision-makers must grapple in the course of negotiating health policy development; such inputs have not always been transparent. Greater accountability in decision making will be supported by the structured frame of discussion offered by a process of facilitated deliberation around an evidence base.

Health resource allocation decisions also may need to go beyond a narrow scientific view of evidence and consider ‘colloquial’ evidence [4] alongside scientific evidence. Deliberative and other consultative processes may usefully be employed to combine these different kinds of evidence to increase the likelihood of achieving ‘sound and acceptable decisions’ (Stern *et al*. in [4]). By including clinical and consumer groups in these processes, it is anticipated that critical experiential knowledge will be added to the scientific evidence collected through systematic review.

Herein, we describe the implementation of methods for broad stakeholder deliberative engagements, a frame for evaluation, and a costing of the process.

## Methods/design

### Overview of the study design

Two case studies were selected for the project: assisted reproductive technologies (*in vitro* fertilization (IVF) and intracytoplasmic sperm injection (ICSI) with specific reference to the safety, effectiveness, and cost-effectiveness related to female age, male age, and cycle rank); and vitamin B_12_ and folate pathology tests. The services/procedures associated with both of these case studies are publicly subsidised under Australia’s universal health insurance scheme, Medicare.

**Figure 1:**
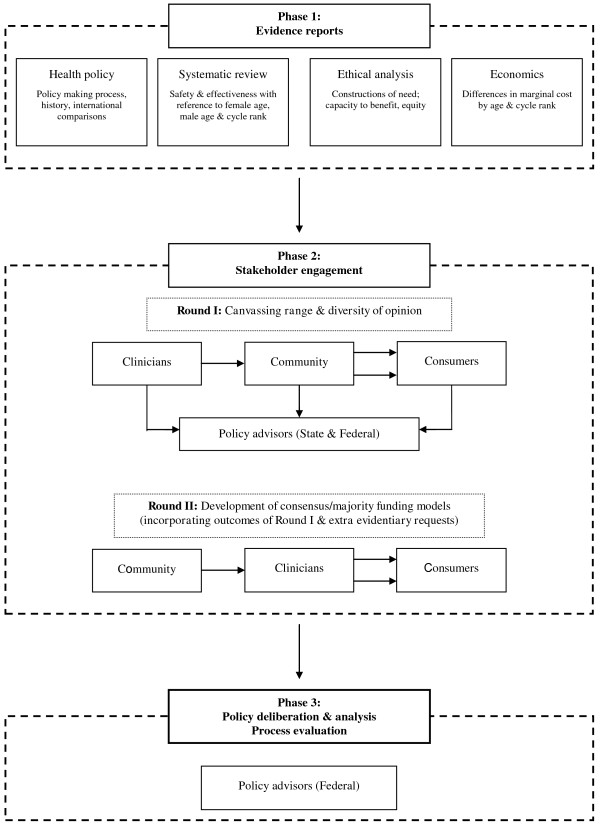
Process of ART case study.

**Figure 2:**
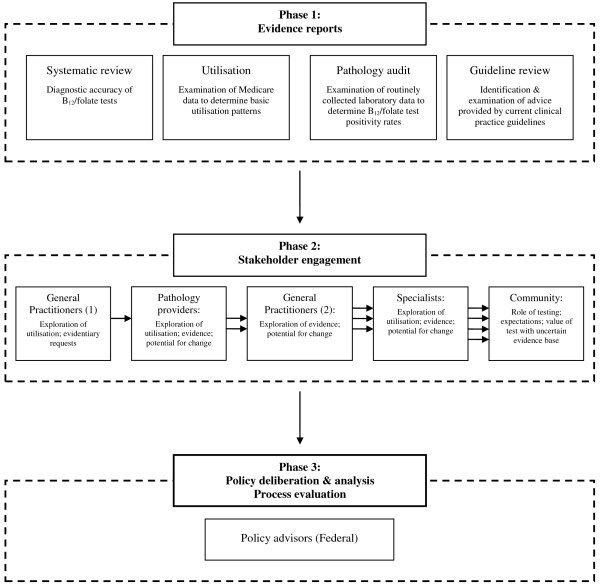
**Process for vitamin B**_**12**_/**folate pathology testing case study.**

The first phase of the project (see Figures 1 and 2) entailed systematic reviews for each case study. These reviews demonstrated the variable effectiveness profiles associated with both assisted reproductive technologies (ART) and B_12_/folate tests [10,11], and collated a wide range of evidence for stakeholders to consider, including ethical and costing analyses.

In the second phase, the findings of these reviews will be presented at a series of deliberative, evidence-informed stakeholder engagements designed to include both motivated stakeholders who have traditionally dominated health policy development (clinicians and consumers) and also the voice of the ‘average citizen’ which is often not heard or excluded from policy decision-making [7]. Each of the stakeholder groups will be actively facilitated towards identifying both shared and dissenting perspectives regarding public funding policy for each of the case studies in response to the evidence presented.

Finally, in phase three, policy advisors will be presented with the results of the reviews, alongside the perspectives of all the stakeholder groups collected in phase two. They will be invited to formulate a disinvestment opinion based on both the scientific and colloquial evidence presented to them, and then to explore their decision-making processes and rationale with reference to the disinvestment context.

The remainder of this paper will focus on project phases two and three as these represent the novel components of the process. A description of the systematic reviews and their findings has been published elsewhere [10,11].

### Selection of case studies

The two case studies of ART and pathology testing for vitamin B_12_ and folate were considered to elucidate an understanding of the broader issues surrounding the development of disinvestment policy [12]. Both were identified as candidates for further assessment under disinvestment initiatives because they met multiple criteria on a proposed identification framework [1], including substantial temporal and geographic variation in usage and early evidence of differential effectiveness across patient subgroups. Additionally, the case studies were considered to engender a variety of different demands on a disinvestment process and were selected in part for their illumination of different potential responses from stakeholders. The ART case study was considered more likely to draw strongly on values-based arguments in the face of relatively low but rising volumes and high cost, while the vitamin B_12_/folate testing case study represented an ostensibly non-controversial, low-cost service, albeit one with high and rising utilization.

To this end, the case studies were selected: to meet the dual goals of research and policy development; to create an innovative approach to shared decision-making; to evaluate the feasibility of participatory processes involving a wide range of stakeholders; and to learn about how these groups perceive disinvestment processes in the context of evidence-informed deliberation.

### Designing evidence-informed deliberative stakeholder engagement

The deliberative engagements for each case study were designed and piloted by a multidisciplinary team (including clinical advisors). Ethics approval for all arms of this study was obtained from The University of Adelaide’s Human Research Ethics Committee.

The case studies were undertaken in a staggered progression to allow for refinement of the process with experience: ART was examined first, followed by the B_12_/folate case study.

In order to avoid capture by vested interests, to maintain a power balance, and to cater for differing levels of expertise, stakeholders were consulted in separate engagements. Potentially partisan stakeholders were not mixed with lay citizens, but the ideas generated by each group were communicated to the subsequent groups to share knowledge and allow cumulative solutions to be constructed.

### Recruitment

Methods of recruitment for the engagement sessions varied between the groups and are detailed in Table 1, with recruitment strategies constructed to take into account the objectives of each deliberative event alongside considerations of feasibility.

**Table 1:** Details of recruitment processes and outcomes

**Stakeholder group**	**Sample size**	**Method**/**s of recruitment**	**Selection and exclusion criteria**	**Demographics**
**Case study 1:assisted reproductive technologies**				
Clinicians	Recruited: N = 19	Nominations from key representative bodies in the field of reproductive medicine*; purposive sampling of key opinion leaders; snowballing	Medically qualified clinicians working in ART and associated fields such as maternal/fetal medicine and neonatology	4 males; 4 females Reproductive medicine: 5/8
	Participated: Round I N = 8	Exclusion criteria:		Other (including obstetrics/gynaecology and neonatology): 3/8
	Participated Round II N = 6		-Participants and/or their partners who had undertaken ART in the 3 years immediately preceding the forum	
			-Participants currently undertaking ART treatment or planning to undertake ART in the foreseeable future	
Consumers	Recruited: N = 32	Purposive recruitment seeking participants with a broad range of ART experiences - advertisement seeking participants placed in The Advertiser (Adelaide’s daily newspaper). Detailed topic and exclusion criteria^‡^	-Females 18 years and older who had undertaken ART treatment, regardless of infertility aetiology	1 male; 8 females
	Participated: Round I N = 9		-Up to 3 males, partners of women who had undertaken ART	
	Participated: Round II N = 7		Exclusion criteria:	
	Reason for exclusion:		-Participants and/or their partners who had undertaken ART in the 3 years immediately preceding the forum	
	Withdrew interest: 2			
	Unable to contact further: 4			
	Unable to attend:1		-Participants currently undertaking ART treatment or planning to undertake ART in the foreseeable future	
	Related to other participant: 1			
	ART undertaken <3 years prior: 12		-An inability to speak and read English	
	Currently undergoing ART: 1		-Pregnant women	
Community	Recruited: N = 25	Random sample of SA population, identified and contacted by independent recruitment company	18 years or older; matched against predetermined stratification criteria for broadly proportional representation of the Australian population^§^	7 males; 7 females
			Exclusion criteria:	Age:
	Participated: Round I N = 14	6 participants had previously participated in a separate citizens’ jury	-Participants currently undergoing ART or who had undergone ART	18-30: 4/14
	Participated: Round II N = 10		-Participants planning to undertake ART in the foreseeable future	31-40: 1/14
	Reasons for exclusion:		-An inability to speak and read English	41-50: 3/14
	Withdrew interest: 5			51+: 6/14
	Missed recruitment deadline: 5		-Pregnant women	Post-tax income:
				<AU$800/week: 7/14
	Did not meet inclusion criteria: 1			>AU$800/week: 7/14
**Case study 2:B12 and folate pathology tests**				
Clinicians: General Practitioners	Recruited: N = 26	Letters of invitation sent to all GPs from 4 South Australian Divisions of General Practice (Adelaide Hills; Adelaide Western; Adelaide Northern’ Yorke Peninsula); respondents selected on ‘first-come’ basis with 2 places reserved for GPs from regional areas	Registered GP working part-time or full-time in active practice in South Australia	9 males; 5 females
	Participated: N = 14			
	Reasons for exclusion: Withdrew interest: 4			
	Quota filled: 8			
Clinicians: Specialists	Recruited: N = 7	Purposive sampling with key practitioners sent letter of invitation; snowballing.	Participants must be registered specialist practitioners (including, but not limited to: geriatricians, physicians, endocrinologists and haematologists) working full-time or part-time in active practice in South Australia	4 males; 2 females
				Speciality:
	Participated: N = 6			General physician: 3/6
	Reasons for exclusion: Withdrew interest: 1			Endocrinologist:1/6
				Haematologist: 2/6
Pathology providers	Recruited: N = 9	Nominations from national public and private pathology interest groups^ǁ^; purposive sampling of key opinion leaders; snowballing	Participants must be working full-time or part-time in administrative, management or laboratory roles in private or public pathology providers in Australia	5 males; 3 females
	Participated: N = 9			Role and practice:
				Public pathology: 2/8
				Private pathology: 5/8
				Management: 1/8
Community	Recruited: N = 16	Random sample of SA population, identified and contacted by independent recruitment company	18 years or older; matched against predetermined stratification criteria for broadly proportional representation of the Australian population^§^	5 males; 6 females
				Age:
	Participated: N = 11			18-30: 3/11
	Reasons for exclusion: Withdrew interest: 5			31-40: 2/11
				41-50: 3/11
				51+: 3/11
				Post-tax income:
				<AU$800/week: 5/11
				>AU$800/week: 6/11

NOTES: ART assisted reproductive technologies; GP general practitioner; SA South Australia.

*including the Australian Medical Association; Royal Australian and New Zealand College of Obstetricians and Gynaecologists; Royal Australian College of Physicians; Royal Australian College of General Practitioners; Fertility Society of Australia.

^†^Many of the reproductive medicine specialists held dual qualifications/clinical roles in gynaecology.

^‡^Copy available from the authors on request.

^§^Stratification criteria based on national Australian Bureau of Statistics data and included: gender (50:50 male:female ratio); age (equal representation from 4 age groups: 18–30; 31–40; 41–50; 51+); and household disposable income (post-tax) (50:50 household income <:>$800.00 per week).

ǁ including National Coalition of Public Pathology, Australian Association of Pathology Practices; Australasian Association of Clinical Biochemists; Healthscope; Sonic; and Primary Health.

Participants in the clinical, consumer, and policy groups were purposively recruited on the basis of their experiential knowledge, stature as opinion-leaders, and role in decision-making processes. Nominations for clinical participants were sought from key stakeholder bodies in the respective fields of reproductive medicine and pathology. Nominees were then approached with a letter of invitation, as were key practitioners in each field.

Consumers (ART case study only) responded to an advertisement placed in the highest-circulating daily newspaper in South Australia. Due to the ubiquity of the test, an appropriate consumer group for the B_12_/folate case study could not be identified.

Policy advisors across portfolios, divisions, and branches (state and federal) directly related to health funding were approached by the project’s Chief Investigators and invited to participate in the evaluative component of the project, with further policy participants identified through snowballing.

Recruitment of community members sought participants descriptively representative of Australian population demographics, although it is acknowledged that such a small sample cannot be completely statistically or politically representative [13]. Pre-determined stratification criteria (gender, age, household income) were applied by an independent recruitment company to a randomly sampled group of South Australian residents, thus avoiding oversampling from older or more economically-advantaged groups.

Sample sizes for the community engagements were based on those advocated for citizen’s juries [14-17], while the other engagements were slightly smaller, reflecting the specificity of recruitment requirements. These were particularly rigorous for the ART case study, because the sensitivity of the topic under discussion required exclusion criteria designed to minimise any potential harm to participants. We felt a duty of care towards consumers who had not been able to achieve a successful live birth because this can be the source of much ongoing distress. The sample sizes were considered optimal to deliver a diversity of opinions while still allowing full and active participation in ‘communicative processes and will-formation’ [18]. From a research perspective, the sample sizes were sufficient to test the logistical and practical components of the engagement design [6], while also evaluating the benefits and drawbacks of the use of deliberative engagements in future decision making beyond the bounds of research.

### Structure of the deliberative engagements

The two case studies differed in their structure for a number of reasons. The ART case study engaged each stakeholder group in two ‘rounds’; this was designed to allow adequate time to address the morally and emotionally complex nature of the topic. It also allowed the opportunity for each group to hear the perspectives of the other stakeholders, and question, incorporate, or discard those views as they deliberated further.

Consumer engagements for the ART case study were approximately four hours long, and held on a mid-week evening over dinner. They included short evidence presentations and a wide-ranging participant deliberation. Sessions were designed to build from participants’ experiences, allowing them to extrapolate to consider broader implications for the healthcare system.

The process for the B_12_/folate case study was streamlined, with general practitioners (equivalent to primary care physicians) the only group consulted twice. With the largest proportion of pathology test requests in Australia arising from primary care [19], it was considered critical to place significant emphasis on interaction with general practitioners in order to ascertain their views on the tests, the evidence, and the potential for change through the lens of primary care. The practicalities of assembling national-level stakeholders across multiple rounds for the purposes of the research precluded a second round of engagement.

The deliberative engagements for clinicians (specialists, general practitioners, and pathology providers) were designed to incorporate their significant experiential knowledge. Four-hour sessions, drawing on existing knowledge and experience of participants, included short evidence presentations and a meal, allowing approximately three hours of participant deliberation. The majority of these sessions were held in the state of South Australia, with only the pathology provider engagement taking place in another state (New South Wales; due to the participants being nominated by their national-level organisation).

The community engagements were held over two days (non-consecutive for ART; consecutive for the B_12_/folate case study), designed to allow time for information sharing, deliberation, and relationship building, acknowledging that the participants were not content experts. Table 2 details the structure for each of the sessions, all of which included a variety of information presentation (adapted for the informational needs of each group), group activities, and deliberation. In all groups, deliberation occurred in a variety of formats, including large and small group brainstorming, small group discussion, and open discussion. Experts who had presented information (including a general practitioner, pathologist (B_12_/folate case study), ethicist (ART case study), and research staff) were available for questioning on points requiring clarification or to further inform, but did not take part in deliberations.

**Table 2:** **Daily agendas for the community engagements**: **assisted reproductive technologies and pathology testing for vitamin B**_**12**_**and folate**

**Assisted reproductive technologies:**	
**Day 1**	
Objectives:	Specific Activities:
Share knowledge regarding health policy in Australia, assisted reproductive technologies and frameworks for decision-making.	Welcome, introductions and orientation (large group)
Explore preliminary responses and construct funding criteria.	Presentations and questions: health policy in Australia, ART safety and effectiveness (large group); costs; ethical frameworks (100 minutes; large group)
	Activity and discussion: International comparisons to identify perceived strengths and weaknesses of different funding models (40 minutes; large group)
	Activity and deliberation: Construct exhaustive list of possible criteria that could be used to restrict funding for ART (large group); refine this list (small groups) and vote on top 5 criteria that should and should not be considered for imposing funding restrictions (individuals) (total 90 minutes)
	Discussion: other information required to construct funding scenarios (30 minutes; large group)
**Day 2**	
Objectives:	Specific Activities:
To determine if the criteria for the public funding of ART should be changed; why; and how	Presentation and questions: information requested after day 1 (15 minutes; large group)
	Presentation and questions: summary of findings from other stakeholders (15 minutes; large group)
	Activity: Construction of funding scenario (45mins; individual/pairs)
	Activity and deliberation: Consensus building on funding scenarios (75 minutes; large group)
	Debrief and close
**Pathology testing for vitamin B**_**12**_**and folate:**	
**Day 1**	
Objectives:	Specific Activities:
To determine what considerations should be taken into account when making decisions about how B_12_/folate pathology tests are publicly subsidised	Discussion: experience and expectations as healthcare consumers (50 minutes; large group)
	Presentation and questions: pathology in General Practice; defining the policy context (75 minutes; large group)
	Discussion: Responding to utilization changes (45 mins; large group)
	Activity and participant questions: General testing epidemiology (60 minutes; large group)
**Day 2**	
Objectives:	Specific Activities:
To determine what considerations should be taken into account when making decisions about how B_12_/folate pathology tests are publicly subsidised	Presentation/questions: B_12_/folate testing (30 minutes; large group)
	Presentation and questions: Rise in level of B_12_/folate testing and stakeholder responses (15 minutes; large group)
	Activity and deliberation: What things should be considered when making decisions about how much we should publicly subsidise B_12_/folate pathology tests? List of factors for consideration (large group); determine 5 most important factors (small group); consensus building activities (large group) (total 150 minutes)
	Debrief and close

In line with the evaluative nature of these engagements, phase three sessions with the policy makers will be structured around a presentation of the evidence base and discussion of the responses from stakeholders, followed by a set of open-ended questions that will allow them to explore the outcomes, strengths, weaknesses, and overall value of the process from a policy perspective. The two-hour roundtable sessions will be held in the Australian Capital Territory and South Australia, and commence with an overview of the project’s outcomes. Participants will be encouraged to engage in an open dialogue regarding each of the specific case studies and a reflection on the nature of disinvestment policy more broadly constructed.

### Evidentiary and other inputs

For both of the case studies, the majority of information presented to the participants was based on systematic reviews of the relevant literature that were specifically commissioned as a component of this research [10,11]. These were supplemented with information modules pertaining to other relevant aspects of health funding decision-making, including: description of the technology/procedure; an overview of health funding policy in Australia (complete with Medicare cost and utilisation data by location and demographics) and elsewhere; economic considerations [20]; and broad ethical frameworks to support decision-making [21] (see Figures 1 and 2).

Prior to the roundtable discussions, clinical participants were provided with a brief summary of the topic and a broad indication of the context for the discussion. At each session, expert members of the research group presented the information modules described above and remained available for questioning for the duration of the evening.

Participants in the consumer engagement (ART case study) were told the topic and context for discussion prior to the engagements, but were not provided with any detailed information. Evidentiary inputs were limited in the first round of engagement, which focussed on international variation in ART funding and previously proposed changes to ART funding in Australia. The second round of engagement included a brief presentation on age-specific safety and effectiveness, alongside the perspectives of other stakeholder groups and the funding models that they had constructed.

Based on multiple reports of lay citizens engaging successfully in technically complex areas of health policy [22-24], this process of community engagement was designed around evidence-informed deliberations, requiring participants to be technically informed on a topic without influencing their deliberation [5]. For the community groups in each case study, the information presented was designed to be accessible to participants with no prior knowledge of the topic areas.

Aware that the topic of ART can engender highly emotional reactions, written versions of the information modules designed to be accessible to a lay audience were distributed one week prior to the engagement session to allow participants the chance to consider their views on the public funding of ART before entering into a potentially evocative group deliberation. This was not considered necessary for the B_12_/folate case study due to its relatively more benign characteristics.

During these sessions there were a number of presentations from expert witnesses with regard to: current Australian health funding policies; safety and effectiveness parameters of the technologies; and economic [20] and ethical considerations [21] (see Table 2). Details of the clinical application of ART were also presented, and the B_12_/folate case study included educative components related to the interpretation of test characteristics (*e*.*g*., diagnostic accuracy as measured by sensitivity and specificity) and clinical aspects of test ordering. The speakers who presented each of these sessions remained available to answer questions during subsequent deliberations.

Policy advisors will be provided with a detailed briefing paper one week prior to the session, providing an overview of the systematic review and summary points from each stakeholder engagement. Brief presentations will be provided by senior research staff, who will remain available to answer questions and provide clarification on any points for the remainder of the engagement session.

### Facilitation

Clinical, community (citizen), and consumer (patient) engagement sessions were facilitated by an independent and neutral facilitator, who was not involved in participant deliberation or questioning of experts (except on points of clarity) and did not provide expert information. The facilitator managed the group interactions, ensuring that all participants had the opportunity to be heard; encouraged in-depth discussion and negotiation amongst the participants; and assisted participants in identifying areas of consensus and persistent disagreement in order to progress the discussion constructively.

Sessions with policy advisors will be facilitated by the project’s Chief Investigators, who will play a dual role in facilitating the evaluative discussion and providing information regarding the process and its outcomes.

Facilitators drew on micro- and meso-level research questions to inform the specific topics of case studies. At the beginning of each session and throughout the discussion, participants were asked to consider specific questions, with participants in the ART case study being asked, for example: ‘Should the criteria for public funding of IVF be changed? If yes, how and why? If no, why?’; and those participating in the B_12_/folate case study: ‘What things should be considered when making decisions about how much we should publicly subsidise B_12_/folate pathology tests?’ Meta-questions regarding the barriers and facilitators to disinvestment more generally and the role of various stakeholders in health policy decision-making were also explored with each group. Supplementary questions varied according to the participant’s role (clinician, consumer, citizen (community), policy advisor) and time was allowed for open, participant-driven discussion.

Members of the community, consumer, and clinical engagement groups were offered a small honorarium for each session they attended in order to cover any expenses associated with their participation. Policy advisors will not be offered an honorarium for their participation, because it is considered that consultations of this nature align with current government activity and thus fall within the remit of their professional roles.

### Data collection and analysis

Given the dual research/policy development objectives of this project, collection and dissemination of both the deliberative and analytical outputs of the stakeholder engagements were considered critical [22]. That is, our analysis is set to generate both a thematic representation of the content of deliberations, as well as more meta-level discussion around the ideological and material implications of the arguments and values articulated.

After obtaining consent from participants, deliberations were audio-recorded and transcribed verbatim by an experienced Hansard reporter (all speech machine typed in full as opposed to shorthand in real time). This occurred contemporaneously for all engagements.

The research component of the project demands that the content of participants’ deliberations be analysed in order to examine the social processes of negotiation and practical reasoning inherent in disinvestment decision-making. As such, the transcripts will be analysed using a synthetic approach to discourse analysis [25], combining the traditions of conversation analysis/ethnomethodology with an interest in broader cultural and historical power relations [26]. This approach allows a focus on the structure of participants’ accounts (how they were put together rhetorically so as to appear credible or persuasive in the deliberative context) as well as their function in justifying broader disinvestment outcomes.

Deliberative outputs of the process will be reviewed and discussed by the research staff with reference to the transcripts, in addition to the notes, lists, and other written outputs produced by the participants. Because pre-formulated options (requiring only a consensus ‘yes/no’ decision) were not offered to the participants, a singular, consensual ‘outcome’ of the deliberation may not be immediately (or ever) evident. For example, in preliminary analyses we see that participants revised their positions over the course of the discussions. This appears particularly evident in engagements with community groups, and requires that the report detailing the deliberative output of this group be endorsed by the participants after the conclusion of deliberation.

### Costs

Table 3 details the expenses associated with the engagement components of each case study (completed at the time of writing). These costs may be variable, and will depend on the purpose, context, and extent of engagement. Although these costs may appear large at first sight, they are small compared to the cost implications of continued use (or otherwise) of the relevant technologies. The costs associated with undertaking the systematic reviews on which the stakeholder engagements are based are not included in this summary, nor is the time of lead study investigators.

**Table 3:** **Summary of expenses associated with stakeholder engagements** (**all costs are in Australian Dollard**, **incurred over 2009 to 2012**)

**Expense category**	**ART case study**	**B**_**12**_/**folate case study**	**Total**
Honoraria	$7,849	$11,231	$19,080
Participant recruitment	$3,653	$3,500	$7,153
Venue			
> Food and drinks (inc. room hire)	$7,293	$9,887	$17,180
> Equipment hire	-	$427	$427
Expert witnesses	$7,040	$9,152	$16,192
Facilitation	$6,978	$3,600	$10,578
> Facilitation associated costs	$5,608	$2,622	$8,229
Stenography	$7,514	$6,913	$14,427
Flights			
> Participant	-	$1,663	$1,663
> Research	$2,474	$2,752	$5,226
Accommodation			
> Participant	-	$160	$160
	$350	$803	$1,153
Project management	$4,800	$4,800	9,600
Staff salaries (administrative support*)	$35,000	$35,000	$70,000
Other operating costs	$1,945	$531	$2,476
**Total**	**$90**,**504**	**$93**,**041**	**$183**,**544**

* Does not include research staffing costs for the development of systematic review evidence, costings and ethical analyses, or supervision time by Chief Investigators.

## Discussion

Drawing on the strengths of health technology assessment and deliberative democratic theory, this program of research attempts to create replicable processes for both governments and private payers to develop transparent, evidence-based policy decisions in the domain of disinvestment.

Systematic review is well-accepted in Australian health policy decision-making, particularly as a component of health technology assessment. We are interested if systematic review will be seen as equally crucial to precede disinvestment decisions, possibly by reducing information asymmetry between policy advisors and clinical and other stakeholder groups.

We hypothesise that the deliberative engagement with the community will be seen to add valuable information to a policy decision, helping to balance the vested interests of single-issue consumer groups. However, it may be that the outcomes of engagement with partisan stakeholders (clinicians and consumers) are viewed with more caution; an assessment of the value of engaging with these groups will be an important outcome of this process.

At the time of writing, the program has been successful in engaging a wide range of stakeholders in constructive and wide-ranging deliberations. Analysis of the varied outputs of the deliberative engagements will contribute to the methodological development around how to best integrate scientific and colloquial evidence for consideration by policy advisors and may contribute to the legitimization of broad and transparent stakeholder engagement in this context.

Embedding disinvestment research more firmly within the established domain of quality and safety may reorientate these discussions towards provision of the most effective care, facilitate more transparent discussions, and help alleviate the incorrect perception that disinvestment is a process of rationing.

From a process perspective, there are clear challenges in recruiting appropriate stakeholder groups, informing deliberations in a neutral manner and supporting active deliberation. Stakeholder groups will vary across technologies and procedures examined, and it is unlikely that a ‘one size fits all’ approach to the undertaking of a process such as this will be appropriate. There remains a role for policy makers to further define the parameters of stakeholder engagement that they will accept as making a legitimate contribution to policy development, and this study hopes to progress this. In doing this though, it is critical that this type of process not be used as a form of ‘market research,’ or to retrospectively justify policy decisions.

This work is situated at the intersection of health policy, health technology assessment, and deliberative democracy. As such, it is theoretically possible to evaluate it from a number of perspectives; the outcomes can be variously defined and evaluated. While the ultimate outcome of this work may appear to be a disinvestment decision for each of the case studies (removal or restriction of funding), this overlooks the fact that a change of such magnitude is reliant on multiple actors and processes outside the bounds of the research sphere; the researcher is not the implementer of policy change. As such, failure to implement an actual disinvestment decision from policy groups should not be considered a failure of this process. However, as this project is designed to contribute pragmatically to policy development (in both the public and private funding domains), it is considered that those actively involved in the development of health funding policy in Australia are in a unique position to evaluate the project’s outputs.

As such, senior policy advisors within state and federal levels of government across multiple relevant portfolios will be approached for their views on the usefulness of the process, its further development, and how the individual components, alongside the deliberative and analytical outputs, might contribute to disinvestment policy development.

We will attempt to uncover from policy advisors what they consider to be the evidentiary requirements for disinvestment processes. They will also be asked to consider—from resource and priority points of view—the desirability and practicality of applying this process across the large number of publicly funded procedures in Australia and the necessary and sufficient conditions that would be required for full or partial disinvestment to be enacted. Views on the processes’ limitations will also be explicitly sought from policy advisors.

While participatory processes hold great promise for their potential to offer a diversity of knowledge and experience to the policy-making process, they do not represent any guarantee of creating a smoother, less contentious policy process. However, when coverage decisions contain significant moral dimensions and dilemmatic elements, decision making may indeed benefit from the experiential knowledge delivered through informed deliberation with engaged stakeholders.

## Abbreviations

ART: Assisted reproductive technologies; EBM: Evidence-based medicine; HTA: Health technology assessment; ICSI: Intracytoplasmic sperm injection; IVF: In vitro fertilisation.

## Competing interests

The authors declare that they have no competing interests.

## Authors' contributions

AGE and JEH conceptualized the ASTUTE Health study and developed the preliminary grant application (methods etc.), with subsequent input from all other Chief Investigators (AJB-M, JRM, HB, JW) and Associate Investigators. AMW made substantial contributions to the study design, acquisition, analysis and interpretation of data and drafting of the manuscript, as did DER, KH, and JMS. JEH and AGE made substantial contributions to the study conception and design, acquisition, analysis and interpretation of data and critical input and revision of the manuscript. AJB-M, JRM, HB, and JW made substantial contributions to the study conception and design, acquisition of data and critical revision of the manuscript. All authors read and approved the final manuscript. The views presented in this manuscript are those of the authors and should not be attributed to The Commonwealth Fund or the Australian Commission on Safety and Quality in Health Care, including officers, directors or staff. All authors read and approved the final manuscript.

## Authors' information

Membership of the ASTUTE Health study group comprises: Chief Investigators Janet E. Hiller, Adam G. Elshaug, Annette J. Braunack-Mayer, John R. Moss, Heather A. Buchan and Janet Wale; Associate Investigators Jonathan D. Karnon, Jackie M. Street, Tracy L. Merlin, Robert Wells, Michael Metz, Paddy Phillips and Peter Littlejohns.
